# Microbially derived polyunsaturated fatty acid as a modulator of gastrointestinal motility

**DOI:** 10.1172/JCI161572

**Published:** 2022-07-15

**Authors:** Yang Xiao, Purna C. Kashyap

**Affiliations:** Enteric Neuroscience Program, Department of Medicine and Physiology, Mayo Clinic, Rochester, Minnesota, USA.

## Abstract

Gastrointestinal (GI) motility requires coordination among several cell types in the intestinal epithelium and the neuromuscular apparatus. A disruption in GI motility was primarily attributed to disruption of this coordinated effort among different host cells, but recent studies have begun to uncover how the products of gut microbiota can alter GI motility by modulating the function of different host cells and the interactions among them. In this issue of the *JCI*, Chen, Qiu, et al. used a reverse translation approach, isolating a *Shigella* sp. — peristaltic contraction–inhibiting bacterium (PIB) — from a cohort of patients with intractable constipation. They identified an ω-3 polyunsaturated fatty acid (PUFA), docosapentaenoic acid (DPA), produced by this *Shigella* variant, as an important driver of constipation using a series of microbiologic, biochemical, and genetic manipulations combined with in vitro and in vivo studies. This finding advances the field, given that production of DPA is rare in the human gut and appears to have a distinct effect on GI physiology.

## Constipation is globally prevalent

Chronic constipation is a common symptom with an overall median prevalence in adults of around 16% and a higher prevalence in women, adults over 60 years of age, and older adults who reside in institutions (1). Constipation can be classified by the Rome criteria, which help categorize patients with gastrointestinal (GI) symptoms. Patients and physicians often regard constipation as a decrease in stool frequency — such as less than 3 bowel movements a week — but Rome IV criteria also include symptoms such as straining, lumpy or hard stool, sensation of incomplete evacuation or anorectal blockage, and manual maneuvers to facilitate defecation as indicators of constipation (2). An important consideration is the presence of symptoms for at least 3 months, with onset of symptoms within the past 6 months, which highlights the chronic nature of the symptoms. While patients with constipation may experience abdominal pain, the presence of abdominal pain at least once a week in association with defecation and a change in frequency or form of stool (with greater than 25% Bristol 1 or 2 stool) is categorized as constipation-predominant irritable bowel syndrome (IBS) (2). Alternatively, constipation can also be defined as (a) normal or (b) slow stool transit constipation or (c) pelvic floor dysfunction/defecatory disorder, based on underlying changes in GI transit or anorectal function.

## Gut microbiota in the pathophysiology of constipation

The etiology of constipation is multifactorial, but recent studies have found a bidirectional interplay between GI transit and gut microbiota. Gut microbial metabolites alter GI transit, and changes in GI transit, in turn, are associated with alteration in gut microbiota composition and function. Furthermore, change in gut microbial function due to delayed GI transit promotes a feed-forward loop that further slows GI transit, highlighting the reciprocating reinforcement of pathophysiological host-microbe interaction (4). GI transit time (time for passage of food bolus thought the GI tract) or bead expulsion time (time for expulsion of a glass bead inserted through the anus) are often used to assess whole gut or distal colon contractility and secretion, respectively, in rodent models. This process requires careful coordination between multiple cell types, including enteroendocrine and goblet cells in the epithelium, enteric neurons and glia, interstitial cells of Cajal (ICC), and smooth muscle and immune cells (5). Typically, studies in preclinical models have focused on the interaction among the different host cells, but recent studies have found that alterations in specific gut microbial metabolites or components can alter GI transit by influencing interaction among host cells.

**Figure 1:**
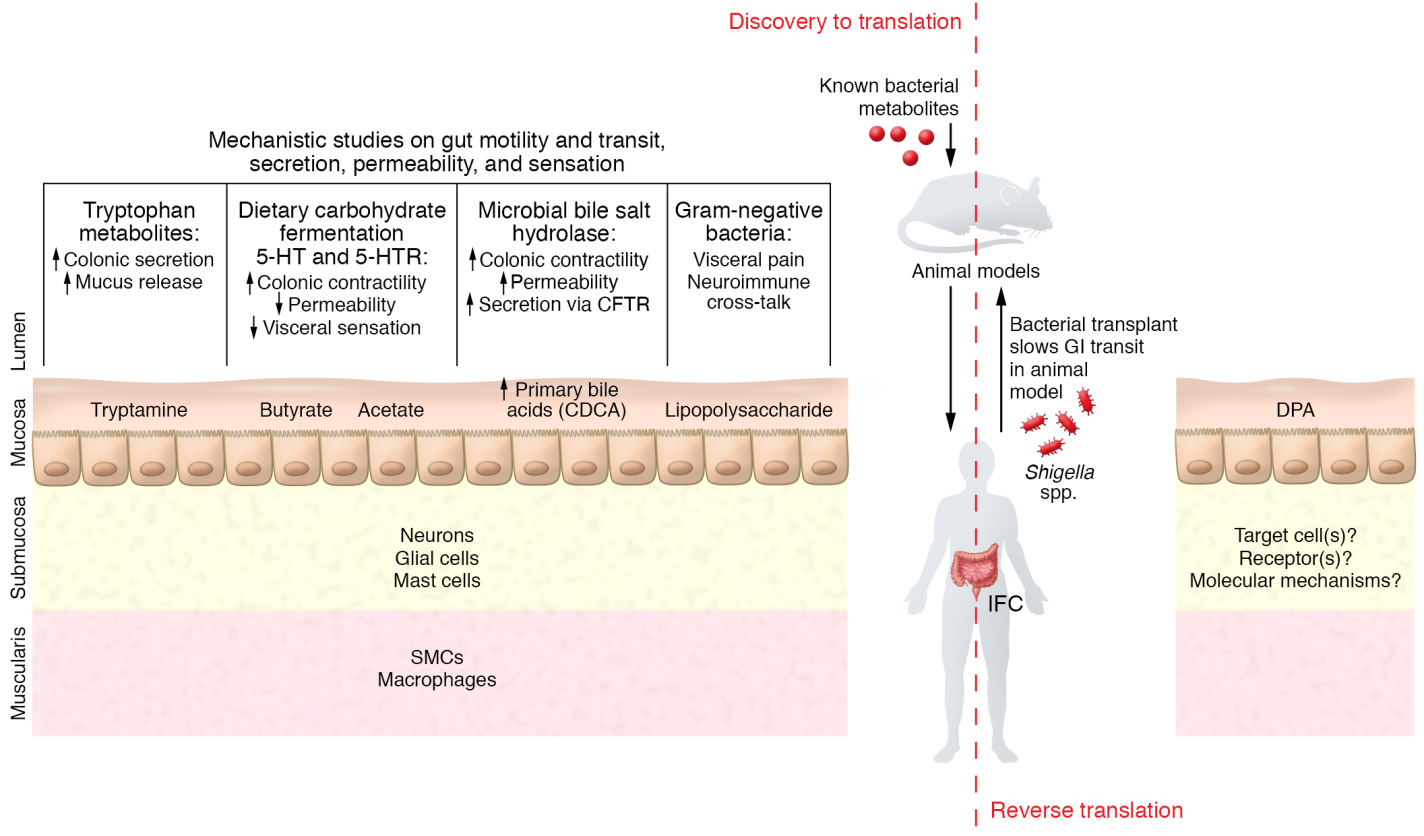
Schematic summary of mechanisms underlying microbial modulation of GI physiology. The conventional, discovery-to-translation approach of studying microbial modulation of GI transit has focused on the effect of known bacterial metabolites, such as short-chain fatty acids and tryptophan metabolites (10–12); cell wall components like LPS; and bacterial functions such as transformation of primary bile acids, on the different cell types (7–9, 15, 16). In contrast, the Chen, Qiu, et al. study used a reverse translation approach, starting with patients diagnosed with IFC and identifying the microbial meditator DPA that prolonged GI transit. Similar to other metabolites, DPA likely alters GI transit by an effect on the enteric nervous system (17). SMCs, smooth muscle cells; 5-HT, serotonin; 5-HTR, serotonin receptor; CFTR, cystic fibrosis transmembrane conductance regulator;

Several studies following the typical discovery-to-translation paradigm have identified microbiota-driven mechanisms that can alter GI transit. These studies utilized an array of preclinical models to determine the role of gut microbiota, such as using antibiotics to disrupt gut microbiota in conventionally raised mice, or comparing conventionally raised and germ-free (GF) mice. The specific effect of microbe(s) is often investigated by colonizing GF or antibiotic-treated mice with individual or a consortia of bacteria or human-derived gut bacteria. The findings from these models are combined with ex vivo (e.g., organ bath studies), culture-based (primary cells or organoids), and omics-based studies to delineate the mechanisms driven by cellular components or metabolites of microbiota. These initial discoveries will need to be corroborated in humans before being developed as therapeutics (Figure 1).

Key findings have emerged from such discovery-to-translation studies. A comparison of GF and conventionally raised mice found that gut microbiota are important for the maintenance of mucosal enteric glial cells (6). LPS, an outer, surface component of Gram-negative bacteria, can affect enteric neuronal survival, increase mast cell degranulation, and influence GI motility by disrupting crosstalk between muscularis macrophages and neurons via the activation of the TLR4/NF-κB pathway (7–9). Fermentation of dietary nutrients results in microbial metabolites such as short-chain fatty acids, including butyrate, that can alter GI transit indirectly, by increasing the synthesis of serotonin (5-hydroxytryptamine; [5-HT]) (10, 11), or directly, by affecting cholinergic neurons in the myenteric plexus. Tryptamine, produced by the microbial decarboxylation of tryptophan, can facilitate GI transit by activating 5-HT4 receptors on intestinal epithelial cells and goblet cells, resulting in increased colonic secretion and mucus release (12, 13). Specific bile acids that enter the colon can increase colonic secretion and contractility; gut microbiota regulate the bile acid species in the colon by deconjugating as well as converting primary to secondary bile acids (14, 15). A recent study found that dietary components interact with the deconjugating capacity of gut microbiota to influence GI transit via a potential effect on enteric glia (16). These studies highlight mechanisms by which gut bacteria might influence GI transit. The next step should involve corroborating the findings in human studies (Figure 1).

## Microbially produced docosapentaenoic acid drives constipation

In this issue of the *JCI*, Chen, Qiu, et al. (17) used a reverse translation approach to identify a bacterial strain and its product that are responsible for slowing GI transit. The authors focused on a patient population with slow GI transit that met Rome IV criteria for constipation and were being considered for surgical intervention after failing conventional medical treatment. They refer to these patients as having intractable functional constipation (IFC) and found the culture supernatant from *Shigella* sp. peristaltic contraction–inhibiting bacterium (PIB) isolated from these patients inhibited spontaneous, but not stimulated, contraction in ex vivo mouse colon and jejunum segments. The authors identified docosapentaenoic acid (DPA, an ω-3 polyunsaturated fatty acid [PUFA]) as the inhibitory metabolite produced by *Shigella* sp. PIB by separating the culture supernatant into polar and nonpolar phases followed by HPLC and LC-MS/MS. The inhibitory effect of DPA was verified in vitro and in vivo and further confirmed with a knockdown strain generated using a CRISPR interference system that targeted the ketoacyl synthase (KS) gene required for generating DPA. Mice colonized with the WT *Shigella* sp. PIB exhibited substantially longer GI transit time, reduced stool water, reduced frequency and amplitude of contraction in ex vivo colon segments, and reduced number of enteric glia when compared with noncolonized mice. Administration of strain-specific phages successfully eliminated the *Shigella* sp. PIB, reduced DPA levels in the stool, and accelerated GI transit time. Mice colonized with the KS knockdown strain (called PIB-KD) showed physiological activity similar to control mice, contrasting with the effects of the WT strain. These findings support a role for DPA in IFC. The authors extended these findings in a separate cohort to determine if the presence of the *Shigella* strain was restricted to IFC; they did not find this strain in the control groups (57 patients with ulcerative colitis, and 26 patients with self-diagnosed constipation but not IFC) (17). A similar reverse translation approach was used in a recent study wherein a role for microbiota-driven purine starvation was identified as a potential mechanism underlying IBS (18).

## Exciting directions

The discovery by Chen, Qiu, et al., that DPA production by a *Shigella* strain underlies the development of refractory constipation, will spur several lines of investigation. First, bacterial production of DPA is rare in the human gut and is predominantly a function of marine bacteria. It is intriguing to consider how these genes were acquired by this *Shigella* strain and if the associated function is prevalent in patients with refractory constipation across the globe. Second, while the authors found a decrease in enteric ganglia, the mechanism by which DPA exerts its effect on GI transit remains to be elucidated. The decrease in spontaneous contraction may be indicative of increased nitrergic neuronal signaling or of a loss of cholinergic neurons. Further, DPA may also affect the mucosa and submucosa; PUFAs have been found to exert biological effects on immune cells, adipocytes, and neuronal cells via GPR120 or GPR40 (19, 20). Third, dietary supplementation of PUFAs has been reported to modulate intestinal and neuronal activities via an effect on gut microbiota (21); Chen, Qiu, and associates found that even high doses of oral DPA had no effect on colonic function in mice, which may be due to proximal absorption in the small intestine, though the DPA levels in stool were not provided from this experiment (17). Alternately, the lack of response to oral DPA may indicate (a) different physiologic effects of dietary versus microbially derived DPA, or (b) the requirement of additional microbial cofactors.

The study by Chen, Qiu, et al. (17) supports a role for bacterially derived DPA in modulating GI transit and adds to the growing body of literature on the role of gut microbial metabolites in modulating GI physiology. This finding is exciting for both the microbiologists, as they investigate the acquisition of this unique function by gut-adapted bacteria, and for physiologists who will be keen to uncover the specific molecular mechanism underlying the effect of DPA. The reverse translation approach used by the authors, while difficult to execute, allows for more direct application in humans and should serve as a model for future investigations that strive to identify microbial mediators of diseases (17).

## Version 1 07/15/2022

Electronic publication
